# Overview and applications of map and model validation tools in the CCP-EM software suite†

**DOI:** 10.1039/d2fd00103a

**Published:** 2022-08-02

**Authors:** Agnel Praveen Joseph, Sony Malhotra, Tom Burnley, Martyn D. Winn

**Affiliations:** a Scientific Computing Department, Science and Technology Facilities Council Didcot OX11 0FA UK agnel-praveen.joseph@stfc.ac.uk martyn.winn@stfc.ac.uk

## Abstract

Cryogenic electron microscopy (cryo-EM) has recently been established as a powerful technique for solving macromolecular structures. Although the best resolutions achievable are improving, a significant majority of data are still resolved at resolutions worse than 3 Å, where it is non-trivial to build or fit atomic models. The map reconstructions and atomic models derived from the maps are also prone to errors accumulated through the different stages of data processing. Here, we highlight the need to evaluate both model geometry and fit to data at different resolutions. Assessment of cryo-EM structures from SARS-CoV-2 highlights a bias towards optimising the model geometry to agree with the most common conformations, compared to the agreement with data. We present the CoVal web service which provides multiple validation metrics to reflect the quality of atomic models derived from cryo-EM data of structures from SARS-CoV-2. We demonstrate that further refinement can lead to improvement of the agreement with data without the loss of geometric quality. We also discuss the recent CCP-EM developments aimed at addressing some of the current shortcomings.

## Abstract 2

Atomic models from cryo-EM show a preference for geometric quality with the expense of agreement with the map data. This study highlights the lack of validation standards and CCP-EM efforts and related developments in this context.

## Graphical abstract

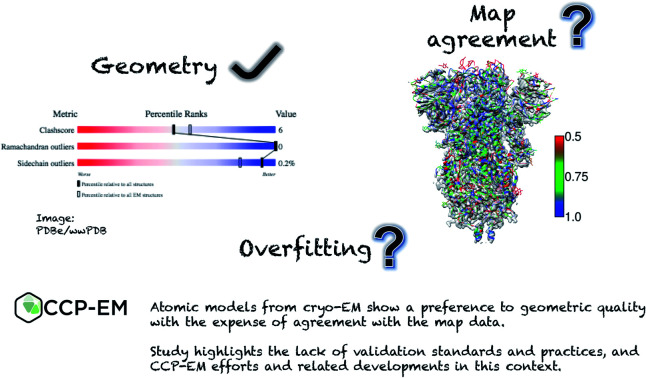

## 1. Introduction and current status of validation tools

The resolution of structures determined using cryogenic electron microscopy (cryo-EM) has improved significantly,^1–5^ resulting in a rapid increase in the number of structures solved. Despite the resolution revolution and associated data explosion, 43.6% of all cryo-EM reconstructions deposited in the EM Data Bank^6^ are in the resolution range of 3–5 Å and about 41% are worse than 5 Å. In the last 5 years, 32% of the reconstructions have been resolved at worse than 5 Å. The average resolution of single-particle reconstructions over the last 5 years is around 5.7 Å.

The need for cryo-EM map and model validation has been recognized over the last decade. Validation spans different aspects including quality of the map or model derived, fit-to-data, overfitting and bias introduced in processing. A validation task force for cryo-EM discussed the community needs and requirements for validation.^7^ This was followed by a number of developments and initiatives focused on validation.^8–10^ The EM data resource map and model challenges^10,11^ have played a very useful role in identifying new requirements and providing datasets for further developments.

An atomic model provides a more interpretable representation of the map reconstruction. However, atomic model building and refinement are increasingly difficult at low resolutions, and hence model validation becomes even more crucial. The geometrical arrangement of atoms in the model is expected to conform to commonly occurring conformations. A number of validation tools developed originally for X-ray crystallography compare the stereo-chemical properties of the atomic model against reference standards (MolProbity,^12^ WHAT-CHECK,^13^ O^14^).

Often, the expected geometry standards are either introduced as part of the function being optimised or as restraints in atomic model building and refinement. Depending on the weights used for these parameters and restraints, one might end up overfitting to expected geometric standards without improving model representation of the data. For example, some refinement approaches overfit the backbone phi/psi angles to the centroid of allowed Ramachandran space leading to ‘unusual’ phi/psi dihedral distribution in the model. Recent studies demonstrate that Ramachandran Z-score^15^ is very useful for detecting such anomalous distributions. Similarly, CaBLAM^16^ was developed to evaluate the quality of the model backbone and detect unusual secondary structure geometries, especially relevant to models built from low resolution data. It is advisable to fix geometry outliers where possible, prior to automated model refinement.^17^

The most common metric used to quantify agreement of the atomic model with the cryo-EM map is the cross correlation calculated either in real space^18–20^ or in different resolution shells in the Fourier space (Fourier Shell Correlation (FSC)).^21^ Several other metrics have also been tested and reviewed in these recent articles.^8,22,23^ With data resolution getting better, multiple methods have been developed to evaluate agreement to map at the residue level.^8,23–27^ The absolute values of most of these metrics vary with the map resolution.^10^

Overfitting to noise in the data is an important factor to consider when trying to optimise model fit-to-map. Over the years several approaches for cross-validation have been proposed to detect overfitting.^21,28–30^ However, the requirement of a sufficiently large independent dataset has been the primary factor limiting the development of a standardised cross-validation approach equivalent to the R-free employed for X-ray crystallography.^31^

Ideally, an atomic model is expected to provide the ‘best’ representation of features resolvable at the data resolution while maintaining a good overall geometry. As cryo-EM data often samples a wide range of resolutions within a single structure, it is important to assess features resolvable at different resolutions and multiple tools are required to evaluate features resolvable at low resolutions. As mentioned above, some of the metrics used for model assessment are intrinsically optimised by automated model refinement approaches and hence multiple and/or independent metrics are recommended for validation purposes. Relative weights for geometry and fit-to-data are often estimated automatically depending on the data quality.^32,33^ However, more often than not, the estimated weights need further adjustment to optimise the fit-to-data without distorting geometry.

Our previous study on a subset of atomic models derived from cryo-EM reconstructions from SARS-CoV-2 revealed a bias in the refinement approaches towards optimising model geometry compared to the agreement with data.^34^ Further automated refinement using REFMAC5 (ref. 33) with a relatively lower starting weight improved the agreement with the maps without significant loss in stereochemical quality. New developments in REFMAC5 (ref. 35) include better weight estimation within the range 0.2 to 18.0, depending on the resolution and ratio of model to map volumes. Here, we use Servalcat to re-refine a large dataset of atomic models (720 structures) of cryo-EM reconstructions from SARS-CoV-2 and discuss the quality of re-refined models. We also highlight other recent developments from the Wellcome Trust UK validation project. We discuss the CCP-EM model validation task developed as part of this project to provide access to multiple validation metrics to assess the geometry and agreement with data, ideally evaluating features resolvable at different resolutions. We also discuss other map and model validation tools available in the CCP-EM software suite.

## 2. Assessment of cryo-EM structures from SARS-CoV-2

### 2.1. Model geometry *vs.* agreement with data

Here we expand the previous study Joseph *et al.* 2022 (ref. 34) to a set of 720 models derived from cryo-EM structures from SARS-CoV-2, available from the Protein Data Bank (PDB) at the end of March 2022. The structures sample a wide range of resolutions from 2.08 Å to 13.5 Å. We evaluated the geometry of these models using MolProbity^12^ and fit to data using FSCavg score.^21^

**Fig. 1:**
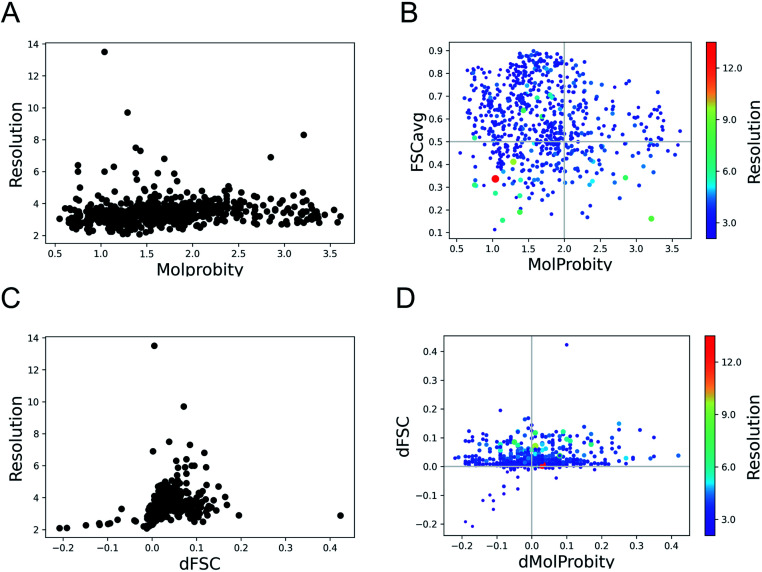
Trends of model geometry *vs.* fit-to-data for 720 deposited SARS-CoV-2 models. (A) The distribution of MolProbity scores *vs.* map resolution. (B) Plot showing FSCavg *vs.* MolProbity scores. The size of the points reflects the resolution of the map, the size-bar on the right shows the scale with respect to resolution. (C) The change in FSCavg scores (dFSC) with re-refinement using Servalcat *vs.* map resolution. (D) The plot of difference (refined − initial) in FSCavg (dFSC) and MolProbity (dMolProbity) scores.

The MolProbity score gives an indication of the quality of the model which is expected to vary with the data quality. There is no clear relationship between the data resolution and model geometric quality as reflected by MolProbity scores (Fig. 1A). 75% of the structures have MolProbity scores better than 2.0, which is comparable to better than 2.0 Å resolution structures.^36^ The mean of MolProbity scores for structures resolved at resolutions better than 3.5 Å resolution is 1.6 while the mean score of structures worse than 3.5 Å resolution is 1.8. This shows that the geometric quality is restrained to a similar extent irrespective of data resolution. Fig. 1B highlights that the geometric quality of the models is not related to their agreement with data, as reflected by FSCavg scores. 31.2% of the structures had FSCavg scores worse than 0.5, reflecting poor agreement with data.

To check whether the automated refinement helps to improve agreement with the data without significant decline in geometric quality, we re-refined the models with 20 iterations of Servalcat.^35^ The fit to data of 94% of structures in the dataset improved after re-refinement. The scores for nearly 20% of the structures improved by more than 5% (dFSC > 0.05) and 5% (35 structures) had more than 10% improvement (dFSC > 0.1). The improvement in FSCavg scores was not correlated with the map resolution (Fig. 1C). However the mean improvement in FSCavg was 6.5% for structures at resolution worse than 5 Å *versus* 2.6% for structures of resolution better than 5 Å. 44% of the dataset had improved MolProbity scores as well while the MolProbity scores of the rest of these structures worsened after re-refinement (Fig. 1D). The drop in MolProbity scores however was not large (less than 0.3 for all but 5 models).

Hence, the fit-to-data could be further improved in a significant majority of these cases without causing a significant loss in geometric quality. The re-refinement with Servalcat also improved the quality of subunit interfaces in the model, as reflected by improvement in PI-scores of 58.6% of subunit interfaces (ESI Fig. S1†). Note that further interactive refinement and error fixes may be required on a case-by-case basis after automated refinement.

As expected, automated estimation of refinement weights using Servalcat improved the fit (FSCavg scores) of a larger portion of the dataset (*i.e.* 94%), compared to improvement of 71% using user-defined initial weight for REFMAC5 in our previous study.^34^ Also 44% of the dataset had better Molprobity and FSCavg scores after re-refinement with Servalcat, compared to 34% from the previous study.

### 2.2. Example 1: model mis-fit with the map

**Fig. 2:**
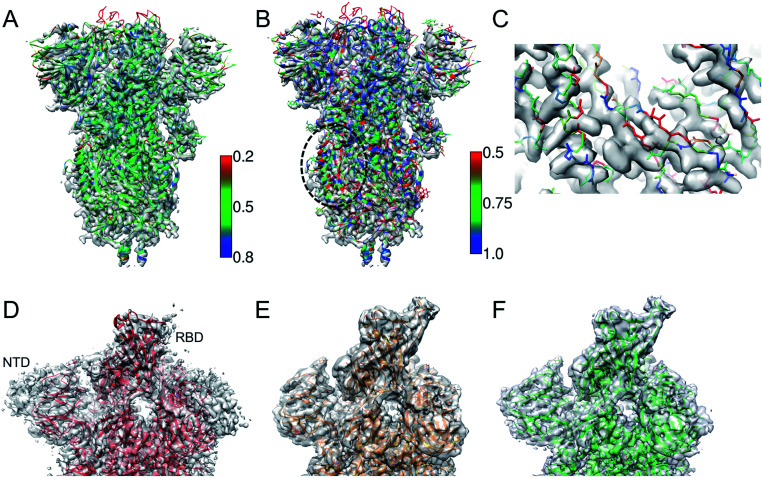
(A) Atomic model of SARS-CoV-2 spike derived from a 3.4 Å resolution cryo-EM map (grey), colored by TEMPy SMOC scores calculated using the CCP-EM software interface. (B) The model colored by the FDR-backbone scores. (C) A segment of the model backbone colored by the FDR-backbone score highlighting areas of mis-trace. (D) The deposited model of the SARS-CoV-2 spike open form (red) (PDB ID: 6VYB) derived from a 3.2 Å resolution map (grey) (EMD-21457). (E) The model with the open RBD coordinates modeled (orange) based on the crystal structure of the RBD bound to the human ACE2 receptor (PDB ID: 6M0J). The LocScale map derived using this model as a reference is shown in grey. (F) The final extended model (green) built with the help of local map sharpening with LocScale (LocScale map shown in grey).

Fig. 2A shows an example of the deposited atomic model of the SARS-CoV-2 spike protein derived from a cryo-EM map resolved at 3.4 Å resolution. The model is associated with a MolProbity score of 1.39 (0 Ramachandran outliers, 1 poor Rotamer, Clashscore: 6.31). However the FSCavg score is 0.21 reflecting poor agreement with the map reconstruction. Per-residue SMOC scores^24^ highlight mis-fit of a significant majority of the residues (Fig. 2A). Low FDR-backbone scores^27^ suggest that the backbone is mis-traced for many of the residues in the model (Fig. 2B). Closer inspection of the model reveals clear backbone mis-traces at several segments of the model (Fig. 2C). This example is just one among several potential cases of poor model agreement with the map. We haven't provided the EMDB and PDB IDs here to maintain anonymity and avoid highlighting issues with this specific deposited model.

This example demonstrates the importance of evaluating model agreement with the map. Although the geometric quality of the model is quite impressive, the model does not provide a good representation of the data due to its poor agreement with the map.

### 2.3. Example 2: partial model

One of the first structures of the SARS-CoV-2 spike protein in the single-subunit open form was solved at 3.2 Å resolution (PDB-ID: 6VYB).^37^ The deposited model had a Molprobity score of 0.77 which is significantly better than that of other models around this resolution (100th percentile). However, a significant part of the Receptor Binding Domain (RBD) of the subunit in the open form was not modelled (Fig. 2D). We used the map processing and model building tools of CCP-EM to extend and re-refine the deposited model. We remodelled the structure of the RBD using the coordinates from the crystal structure of the RBD bound to the ACE2 (angiotensin converting enzyme-2) receptor (PDB ID: 6 M0J^38^) as a reference (Fig. 2E). We used the model to locally scale the map for optimal sharpening, using LocScale.^39^ LocScale maps are useful to detect features especially in areas of lower local resolution where global sharpening might have resulted in broken or noisy density. The locally sharpened map shows features of the RBD domain.

We optimised the fit of the modelled RBD domain using real-space refinement in Coot.^40^ The LocScale map also showed additional features corresponding to the N-terminal domain (NTD) and the C-terminus. Using related structures solved at higher resolutions (PDB IDs: 6VXX and 5X58) as guides, we traced additional residues in the locally sharpened map in Coot. In an iterative process, extended models were then used to make new LocScale maps. In the end, we were able to extend the model by 151 residues (Fig. 2F). Outliers (Ramachandran, Rotamer and CaBLAM) were fixed in Coot where possible. The REFMAC5 interface in CCP-EM^33^ was used to refine the extended and fixed models against the deposited map (not the locally sharpened map, as recommended). ProSMART restraints^33^ were used in refinement when higher resolution related structures were available. The extended model had a MolProbity score of 1.56 and the FSCavg improved slightly from 0.54 to 0.55 (local correlation from 0.86 to 0.88). The extended model is available from the Coronavirus Structure Task Force^41^ repository (https://www.github.com/thorn-lab/coronavirus_structural_task_force/tree/master/pdb/surface_glycoprotein/SARS-CoV-2/6vyb).

In this case, the open RBD and part of the NTD are relatively less resolved compared to the core of the spike. The locally sharpened map however enhanced features in these parts of the map, enabling extension of the model. There is an ongoing debate whether to build models in low-resolution areas of the map or if an ensemble of models (rather than a single model) should be deposited to represent the local variability.^42^ In the case discussed above, the extension of the model provides additional information on the exposed structural segment of the RBD which forms an interface with the ACE2 receptor and antibodies. Hence the modelled segment at low resolution is useful but the level of interpretation should be based on the features resolvable at that resolution.

## 3. Model and map validation in CCP-EM

### 3.1. UK EM validation initiative

To address the validation needs of the community and develop new software, pipelines and training resources, the Wellcome Trust funded EM validation project was set up in 2018 across six different sites in the UK. The consortium addressed some of the concerns raised by the original validation task force,^7^ while also considering how the resolution revolution had changed the nature of typical cryoEM studies. A number of approaches and tools were developed as part of this project, covering assessment of 3D maps against raw data (unpublished); map symmetry estimation (ProSHADE);^33^ automated validation of deposited maps and models (EMDB validation analysis^43^); model interface quality (PI-score^43^); map–map agreement and difference (EMDA^44^) and model–map agreement (FDR backbone score,^27^ EMDA,^44^ 3D-Strudel,^45^ EMDB validation analysis^43^). The majority of these tools are now distributed as part of the CCP-EM software package (v1.6). Below we discuss the CCP-EM model validation task developed as part of this project that integrates multiple validation tools.^34^

### 3.2. Atomic model validation task in CCP-EM

The atomic model validation task (validation: model) in CCP-EM provides an interface to access multiple tools and complementary metrics that evaluate the geometry of the model and fit-to-data.^34^ The aim is to develop this further to integrate other tools to assess features of the map resolvable at different resolutions. Tools for assessing the map quality are not included in this task, but can be found elsewhere in the CCP-EM software suite (discussed below).

The current implementation provides access to MolProbity (various stereochemical checks^12^), CaBLAM (backbone Cα geometry^16^), PI-score (subunit interface quality^46^) and JPred4 (agreement with sequence-based secondary structure prediction^47^). To quantify global agreement with data, REFMAC5 (model–map FSC^48^) and TEMPy (CCC and other real-space scores^22^) can be used. To evaluate per-residue fit, TEMPy (SMOC score^24^) and FDR backbone score (identify mis-traced residues^27^) are provided. The details of the validation tools currently available through this task are discussed in detail in ref. 34. Multiple validation tools not only add more confidence to some of the issues detected but also work in a complementary way by identifying unique issues. Multiple issues in the same structural neighbourhood usually point to more serious errors and often fixing one or more of them can help resolve others in their vicinity. The results from complementary validation tools are collated and sorted to highlight specific structural regions with the most serious issues (clustered by spatial proximity). The results are also linked to Coot^40^ where the issues can be fixed interactively and flagged as complete as and when each residue is fixed by the user.

### 3.3. Other model validation tools

The Privateer task^49^ can be used to validate individual monosaccharide conformations in the atomic model, check whether the modelled carbohydrate atomistic definitions match dictionary standards as well as output multiple helper tools to aid the processes of refinement and model building.

3D-Strudel^45^ scores how well the map features around a certain residue resemble those observed in other structures at a similar resolution, and suggests alternative interpretations (residue types) of the map where the agreement is poor. It can thus identify register errors in model building.

The TEMPy Diffmap task identifies mis-fitted residues by calculating the difference between the experimental map and the theoretical map derived from the atomic model.^50^ This tool can also be used to detect conformational or compositional differences between two experimental maps.

### 3.4. Map validation tools

cryoEF allows a rapid quantification of the particle orientation distribution based on its ability to provide uniform resolution along all directions of the reconstruction.^51^ The method also predicts optimal tilt angles to achieve a more uniform information coverage.

The map to MTZ CCP-EM task applies an array of global sharpening factors for assessment of a post processed map. A Wilson plot is displayed, allowing inspection of potential pathologies arising from over-sharpening,^52^ and the task is linked to Coot for visual inspection.

The ProSHADE task^33^ allows identification of symmetry, given a map or an atomic model. ProSHADE can identify the point group of a map, and hence is useful during deposition as well as during molecular visualisation.

The confidence map task uses the false discovery rate (FDR) approach^53^ to quantify the confidence at each voxel for distinguishing molecular signals from the background. It can detect weak features in the map based on the statistical significance estimate.

EMDA is a toolkit with a range of functionalities for comparing either an atomic model against a map or multiple maps.^44^ The toolkit includes map–model and map–map local correlation, map–map superposition and map magnification correction. EMDA is currently distributed with the CCP-EM software suite and accessible through the command-line.

We are working on integrating tools that are part of the EMDB validation analysis.^43^ This will provide access to different validation tools used by EMDB to evaluate deposited maps and models. Hence, the user can assess their maps and models and fix any issues prior to deposition.

## 4. Application of the CCP-EM validation task to SARS-CoV-2 structure interpretation

Owing to the rapid response of the research community at the onset of the pandemic, there is a wealth of structural and sequence data for SARS-CoV-2. We developed the CoVal service (https://coval.ccpem.ac.uk, manuscript in preparation) to connect data on amino acid replacement mutations, from genomes of the SARS-CoV-2 virus sequenced from human host isolates, with structural data held in the PDB and EMDB. One of the main aims behind the development of this database is to provide indicators on the reliability of structures and their interactions, through the use of quality metrics that are established in the structural biology community. Thus the functional implications of observed mutations should take into account available structural data, but also the reliability of this data. CoVal provided the following details:

(1) Map the mutations onto 3D structures of viral macromolecules determined by cryo electron microscopy and X-ray crystallography.

(2) Link to external resources on protein domain and function annotations.

(3) Visualise the site of mutation on the 3D structures.

(4) List contacts involving the mutation site based on a selected structure.

For crystal structures, we fetch the validation metrics from PDBe using the REST API (https://www.ebi.ac.uk/pdbe/api/doc/search.html) for programmatic access. For the cryo-EM structures, we use the more extensive set of model validation tools implemented in the CCP-EM software suite (https://www.ccpem.ac.uk/)^54^ to calculate multiple metrics that evaluate the geometry of the model and the fit-to-data.

**Fig. 3:**
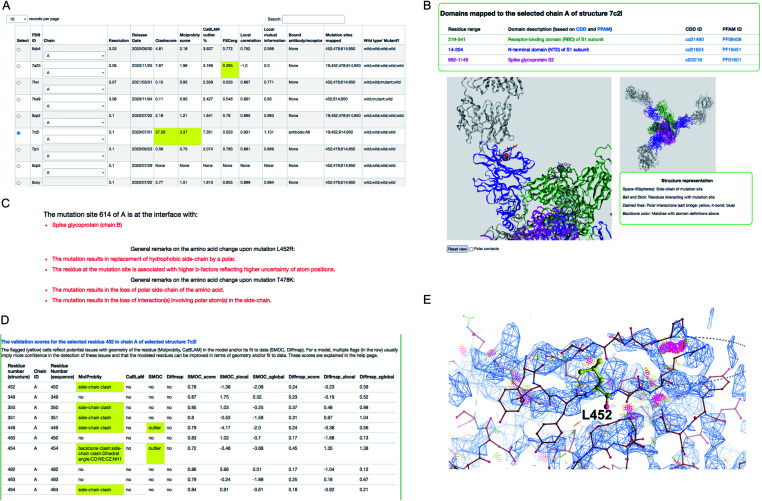
Structure mapping and visualisation. (A) An example of the result retrieved from a search of structures where a selected mutation cluster (associated with the delta variant) could be mapped. For each structure, a set of validation scores are provided to highlight the overall geometric quality and agreement with experimental data. Low scores are highlighted in yellow. (B) Visualisation of mutation site(s) mapped on the selected structure, using the NGL applet. The residues at the mutation sites in the cluster are shown in space-fill representation and the residues in the neighbourhood interacting with these are shown in ball and stick representation. (C) General remarks on one of the mutations in the cluster: D614G, L452R and T478K. (D) Per-residue validation scores covering the mutation site and its neighbours. Cells highlighted in yellow flag low scoring residues. (E) Clashes around L452 (serious clashes shown as pink clusters) identified using Molprobity and rendered in Coot.

We demonstrate an example of mutation search for the spike protein based on genome samples from the UK to highlight the use of the CoVal database. Upon the search, the structure summary page provides a table of metrics appropriate to the experimental technique used, with poor scores highlighted (Fig. 3). Users can select a model based on the resolution and/or the validation scores and choose one of the chains where the mutation(s) is mapped.

For this example, we selected the structure PDB ID: 7c2l from the list of search results. The mutation site(s), its structural environment and polar contacts can be visualised on the selected structure and chain using the NGL applet^55^ (Fig. 3B). In Fig. 3B, the mutation sites are shown as space filled spheres and their structural interactions are shown in ball and stick and the bound antibody is shown as grey ribbon. PFAM and CDD domain definitions are used to annotate the chains in the model and the backbone of each chain is colored using unique colors for each domain. We also provide function annotations for each chain in the model. This includes annotations retrieved from PDBe^56^ and mappings to UNIPROT^57^ and GO.^58^ To provide further guidance, we include general remarks on the effect of mutation on the physico-chemical nature of the amino acid, and whether the mutation site is at the interface with the receptor or antibody or another subunit (chain) in the model (Fig. 3C). The interactions involving mutation sites in the selected structure are listed under the interactions tab.

For cryo-EM structures, we provide multiple validation metrics to highlight any potential errors or ambiguities associated with the model at the mutation site and the interacting residues, reflecting stereo-chemical quality and agreement with experimental data. Fig. 3D shows an example (PDB ID: 7c2l) of various validation metrics which are provided for the mutation site (L452) and its structural interactions. Residues associated with low validation scores (highlighted in yellow) are less reliable compared to others, and hence the user has to be cautious with interpretations based on the atomic details of this residue in the selected model. The clashes in and around the mutation site L452 are highlighted in Fig. 3E suggesting that the atomic coordinates at this site are potentially less reliable for downstream interpretation.

## 5. Discussion and perspectives

Our assessment of cryo-EM structures from SARS-CoV-2 suggests that refinement approaches tend to preserve the stereochemical quality irrespective of the data resolution. Although model geometry may be favoured at low resolutions due to low information content associated with the data, care should be taken to ensure the model is in good agreement with the resolvable features in the map. The quality of the fit to data appears to vary in a non-systematic way, suggesting a wide variability in how refinement tools are applied. To this end, there is a need for validation tools that evaluate the quality of low-resolution features of a model and their agreement with the map.

Further refinement of SARS-CoV-2 structures with Servalcat improved the agreement with maps without significant loss of geometric quality. In fact, the geometry also improved in nearly 44% of the cases and the drop in MolProbity scores for the rest of the cases was not large (less than 0.3 for all but 5 models). The improvement in fit to the maps was not correlated with the data resolution suggesting no clear trend for overfitting to geometry as the resolution worsens. Apart from the estimated or user-defined refinement weights, other user-defined parameters and restraints used in refinement, and the initial fit of the model in the map also influence the final geometric quality and agreement with the map. In this context, efforts like CERES^59^ and extension of PDB-REDO^60^ for models derived from cryo-EM will be important.

Clearly, there is a need to report metrics reflecting agreement with the map alongside geometry evaluations. EMDB has been developing a resource for validation analysis of deposited maps and models where multiple metrics for evaluating model agreement with the map are included.^43^ This will help downstream users of the deposited maps and models to detect reliable areas of the model to base their interpretation on. The model challenges organised by EMDataResource are another useful initiative in this context.^10^ A number of metrics have been proposed to evaluate local model agreement with the map, some of them shown to work in a complementary manner.^10,27^ As in the CCP-EM validation task,^34^ the use of multiple metrics helps to detect a range of potential issues and evaluate different features of the model in a complementary way. We plan to expand this to include tools that evaluate low-resolution features in the model and their agreement with the map.

Building atomic models from cryo-EM reconstructions is increasingly common given the improvement in data resolution. Last year, 2894 of 4483 entries (∼65%) released in EMDB had associated atomic models (https://www.ebi.ac.uk/emdb/statistics/emdb_entries_pdb_models). Hence it is crucial that structural biologists adopt standard practices for model building and refinement, and report validation metrics to reflect the stereochemical quality, fit-to-data and any test for overfitting (to noise in the data) where possible. Ideally, the field needs to work together to agree on a fit-to-data/cross-validation metric, equivalent to ‘Rwork’/‘Rfree’ in X-ray crystallography, that is simple and universally-recognised. It may be, however, that multiple complementary metrics are required, as discussed above. In this context, CCP-EM organises the Icknield workshop every year focused on training users on tools for model building and validation, and supports other workshops on best practices. Cryo-EM map and model validation is an area of development, rightly recognised and supported by different community developments and initiatives across the world.

The pipeline underlying the CCP-EM validation task is used to evaluate all cryo-EM structures from SARS-CoV-2 and the results are provided *via* the CoVal database. In the future, we plan to expand the validation task to provide access to multiple and complementary validation tools that work across a range of resolutions and evaluate different model features. Robust application of validation relies on good data management, and therefore the validation task will utilise the recent development of the Pipeliner framework in the CCP-EM suite which tracks data and metadata of all jobs that are run (or imported). In the end, processing workflows, validation and deposition are closely linked activities.

## Author contributions

APJ, SM, TB and MDW designed the study and drafted the paper. APJ and SM did the data integration and analysis, and developed CoVal.

## Conflicts of interest

The authors have no conflicts of interest to declare.

## Supplementary Material

FD-240-D2FD00103A-s001
