# Supplementary material for: Overview and applications of map and model validation tools in the CCP-EM software suite
Source: Faraday Discuss. 2022 Aug 2;240:196–209. doi: 10.1039/d2fd00103a (PMC9642004; doi:10.1039/d2fd00103a)
Supplement: FD-240-D2FD00103A-s001 [file FD-240-D2FD00103A-s001.pdf]

Electronic Supplementary Material (ESI) for Faraday Discussions.
This journal is © The Royal Society of Chemistry 2022

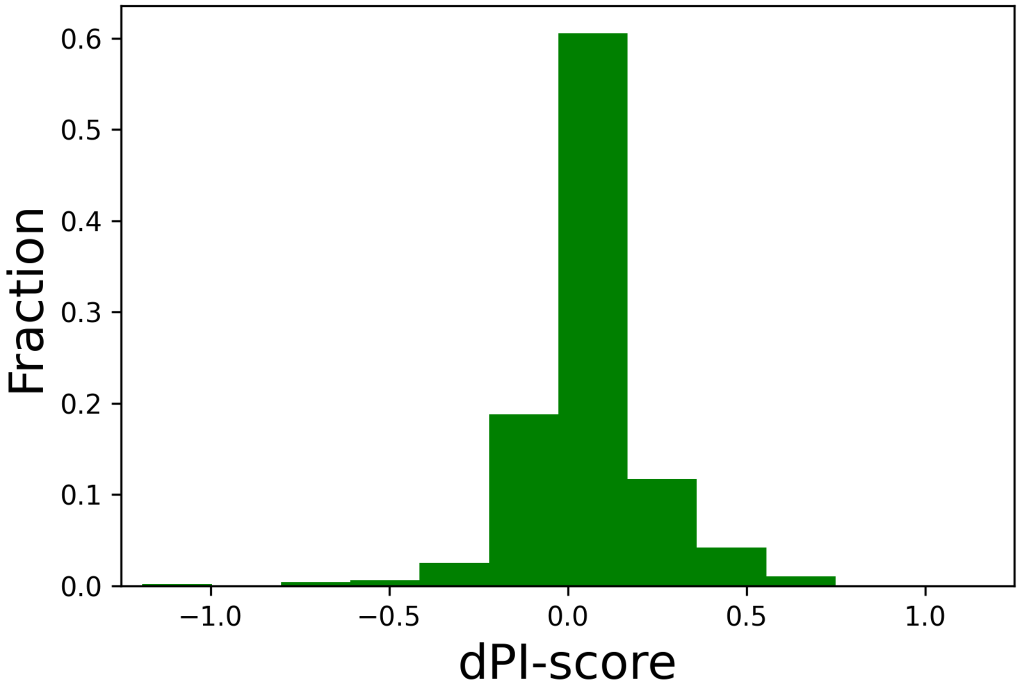

Suppl Figure 1. The distribution of change in PI-scores (dPI-score) of the subunit interfaces in the models re-refined using Servalcat (with respect to that of the deposited models).
